# Development of an integrative learning program for community dwelling old people with dementia*

**DOI:** 10.1590/1518-8345.4794.3486

**Published:** 2021-11-19

**Authors:** Thomas Kwok Shing Wong, Yang Yunhua, Chen Jinghan, Carmen Ka Man Lee, Zhou Ying, Jiang Liping, Tang Qiubi, Joanne Wai Yee Chung

**Affiliations:** 1 Guangzhou Medical University, Guangzhou, Guangdong, China.; 2 Guangzhou University of Chinese Medicine, Guangzhou, Guangdong, China.; 3 Hong Kong Nang Yan College of Higher Education University, Hong Kong, China.; 4 Will Way Wellbeing RD Ltd, Hong Kong, China.; 5 Tung Wah College, Hong Kong, China.; 6 Xin Hua Hospital Affiliated to Shanghai Jiao Tong University, Shanghai, China.; 7 The Education University of Hong Kong, Hong Kong, China.

**Keywords:** Dementia, Neuroplasticity, Learning, Psychiatric Nursing, Community Health Services, Nursing Methodology Research, Demencia, Neuroplasticidad, Aprendizaje, Enfermería Psiquiátrica, Servicios de Salud Comunitaria, Investigación Metodológica en Enfermería, Demência, Neuroplasticidade, Aprendizagem, Enfermagem Psiquiátrica, Serviços de Saúde Comunitária, Pesquisa Metodológica em Enfermagem

## Abstract

**Objective:**

to develop an integrative learning program for people with dementia.

**Method:**

a methodological study was conducted using Delphi technique to develop the learning program, followed by a feasibility test. An expert panel was invited to develop the integrative learning program based on the neuroplasticity and learning framework. A feasibility test was conducted to evaluate the implementation of the program in two centers after the training of personnel who run the program. Verbatim transcripts of case conferences were coded, analyzed, and collapsed into themes and sub-themes by consensus.

**Results:**

there was no indication for content modification during the period of program implementation. Qualitatively, the participating older adults showed improvement in communications, emotions, connectedness with self and others, and well-being.

**Conclusion:**

the integrative learning program was uneventfully implemented with promising results. The program is ready for full-scale research on its efficacy in multiple centers to obtain more robust evidence.

## Abstract (es)

**Objetivo:**

desarrollar un programa de aprendizaje integrador para personas con demencia.

**Método:**

se realizó un estudio metodológico empleando la técnica Delphi para desarrollar el programa de aprendizaje, seguida de una prueba de viabilidad. Se invitó a un panel de expertos a que desarrollara el programa de aprendizaje integrador sobre la base del marco de la neuroplasticidad y el aprendizaje. Se realizó una prueba de viabilidad para evaluar la implementación del programa en dos centros después de haber capacitado al personal que dirige el programa. Las transcripciones literales de los debates de casos se codificaron, analizaron y resumieron en temas y subtemas por medio de consenso.

**Resultados:**

no hubo ninguna indicación para modificar el contenido durante el período de implementación del programa. En forma cualitativa, los adultos mayores que participaron del programa evidenciaron mejoras en la comunicación, las emociones, la capacidad de conexión con ellos mismos y con los demás y el bienestar.

**Conclusión:**

el programa de aprendizaje integrador se implementó sin inconvenientes con resultados prometedores. El programa es apto para ser sometido a una investigación a gran escala con respecto a su eficacia en varios centros para obtener evidencia más sólida.

## Abstract (pt)

**Objetivo:**

desenvolver um programa de aprendizagem integrativa para pessoas com demência.

**Método:**

foi realizado um estudo metodológico com a técnica Delphi para desenvolver o programa de aprendizagem, seguido de um teste de viabilidade. Um grupo de especialistas foi convidado para desenvolver o programa de aprendizagem integrativa com base no *framework* da neuroplasticidade e da aprendizagem. Um teste de viabilidade foi realizado para avaliar a implementação do programa em dois centros após treinamento do pessoal que dirige o programa. Transcrições literais de conferências de casos foram codificadas, analisadas e agrupadas em temas e subtemas por consenso.

**Resultados:**

não houve indicação de modificação de conteúdo durante o período de implementação do programa. Qualitativamente, os idosos participantes apresentaram melhora nas comunicações, emoções, conexão consigo e com os outros e bem-estar.

**Conclusão:**

o programa de aprendizagem integrativa foi implementado sem intercorrências com resultados promissores. O programa está adequado para ser submetido a pesquisas em grande escala relacionadas com sua eficácia em vários centros a fim de obter evidências mais robustas.

## Introduction

Caring for the elderly with dementia is a global challenge. About 5% of the world’s elders (47 million) suffered from dementia and it was estimated that this will rise to 75 million in 2030 and 132 million by 2050^(1)^. In other words, there will be one new case of dementia diagnosed globally every 3 seconds. The severity of the problem can be seen by taking China as an example because it has the largest population with dementia in the world^(2)^. The prevalence of senile dementia (among people aged 65 or above) was rising, from 5% in 2013 to 5.56% in 2017. Taking the Chinese Mainland, Hong Kong, and Taiwan together, it was reported that the number of people aged 60 or above suffering from dementia was as high as 9.48 million in 2018^(3)^. The estimated measures of prevalence of dementia (estimated population) in northern China, central China, southern China, western China, Hong Kong, and Taiwan were 5.5% (3.52 million), 5.2% (3.79 million), 4.8% (1.48 million), 7.2% (0.69 million), 7.2% (0.07 million), and 6.0% (0.15 million) respectively^(3)^. Since China has comparatively quite high dementia prevalence rate, its huge population size will bring huge number of dementia patients with heavy burden on the community. In view of this global problem, the WHO has considered dementia as a global public health priority^(4)^ and taken measures to help countries to contain the problem.

The number of healthy years [disability-adjusted life years (DALY)] lost is tremendous as dementia is the fifth leading cause of death globally. It was reported that about 28.8 million [95% uncertainty interval (UI) 24.5-34.0] DALYs lost were attributed to dementia^(5)^. Dementia also has devastating impacts on sufferers’ families and friends. Family members are often the main caregivers for people with dementia. It is an unpaid and round-the-clock job that causes not only physical and psychological exhaustion, but also a huge financial burden. It was reported that the loss of wages for being unpaid caregivers at home was forecasted to increase from $5 billion Canadian dollars in 2008 to $55 billion Canadian dollars in 2038^(6)^. Besides the wage loss, there are also high intangible costs to caregivers in taking care of people with dementia as stress, fatigue, depression, and anxiety set in.

From a societal perspective, the impact of dementia on social healthcare expenditure and demand for nursing services cannot be underestimated as well. As reported, the total global expenditure on dementia treatment in 2015 was US$818 billion^(7)^. It was projected this will increase to US$2 trillion by 2030. Similarly, another study estimated that the annual economic losses caused by dementia in mainland China alone was 83.5-97.4 billion Yuan, and the consumption of cognitive related health services reached 51.3-59.8 billion Yuan a year^(8)^.

The pathogenesis of dementia is complex and scientists are still unraveling its myths from different perspectives, including physiological mechanism of the disease, neurology, behavioral performance, and other associated aspects^(9-10)^. Up to now no conclusion has been reached and the most controversial debate of all is whether dementia is caused by the dysfunction of the neural circuits in the brain^(11)^ or the malfunction of the cerebrovascular system^(12)^. Supporters of the dysfunction in the brain’s neural circuits believe that the harmful signals of neuritic plaques and nerve fiber entanglement in certain areas of the brain cause the gradual degradation of the brain’s cholinergic system, so they are more inclined to use galantamine drugs to enhance the functions of the sufferers’ cholinergic system^(13)^. On the other hand, for those who believe it is the dysfunction in the cerebrovascular system, they believe that high homocysteine affects the blood vessel systolic reactivity of the cerebral artery which in turn leads to cognitive decline and neuro degeneration, so people are advised to take folic acid and vitamin coenzyme to reduce homocysteine levels^(14)^. However, the current pharmacological approach can only delay the progress of the disease. Drug therapy does not cure dementia^(1,4)^.

In recent decades, scientists and clinicians have been exploring various non-pharmacological treatments for dementia to reduce disability, alleviate and/or manage behavioral and mental symptoms so as to improve the quality of life of the affected people and their caregivers^(2,6,15)^. So far, no effective treatment has been identified^(1,4)^.

At present most, if not all, care models adopt a biomedical approach to manage dementia. Most dementia programs are western medicine oriented, e.g., reality orientation, reminiscence therapy, multisensory stimulation, daily life skills training, and music therapy; complementary approach such as inclusion of Chinese medicinal approach has yet to be adopted.

By taking these experiences into consideration, the team attempted to apply the concept of neuroplasticity and learning to dementia care on the premise that people have the capacity to learn new things through repeated practices. In other words, they can rebuild their capacity gradually through the process of slow stream rehabilitation.

Recent studies supported that the brain is plastic^(16-17)^, which means brain cells can change their structure and functions according to the conditions required. *In vivo*, studies showed that the physical brain structure of mice changed through what they did in their daily experiences in enriched environments^(18)^. All brain cells, including those in damaged brains, have neuroplasticity^(19)^ and especially adult neurons are capable of neurogenesis^(16)^. The adult brains have huge latent plasticity and it is believed that repeated practice can lead to re-organization of the cerebral networks which can enhance functional performance through intense training^(20)^.

Learning is a process of acquiring skills, knowledge, attitude and values. The “learnt” experiences would mold the brain through neuroplasticity. Thus, neuroplasticity is vital to learning as new neurons are formed in the hippocampus and cerebellum of the adult brain through neurogenesis, and thus, new memories are created and older memories may be modified^(21)^. The brain can be rewired just by learning, thinking, and practicing. One way to achieve the change is to activate learning through goal setting and practices in a positive mindful manner in the pursuit of rewards while the brain is creating new paths^(16-17)^; and it is always desirable to have a positive environment for neuroplasticity to emerge and learning to take place, when the medial pre-frontal cortex is associated with a healthy, happy, and positive attitude which can be brought about by coming back to the present moment through mindfulness, a way to exercise the pre-frontal cortex^(22-23)^. To further enhance the change, learning, an application of mindfulness, has been shown to increase the flexibility and attention in learning^(24)^ and the learners’ connectedness to the surroundings^(25-26)^.

To bridge the knowledge and practice gap in current dementia care, we determined to develop an integrative learning program based on the concept of neuroplasticity and learning. It is hoped that the program will help old people with dementia manage their symptoms using a transdisciplinary approach. We can learn new things (e.g. skills, emotions and cognition) in the presence of innate neuroplasticity. The learning can be conducted in combination of group and personal contact. Group learning allows interaction and stimulation while person-centered learning accommodates individual needs. To have learning to occur, we need to repeatedly practice which optimizes our neural networks based on the neuroplasticity. Therefore, the aim of the study was to develop the integrative learning program for people with dementia.

## Method

This was a methodological study in which an integrative learning program based on the neuroplasticity and learning framework was developed by an expert panel using the Delphi technique, followed by a feasibility test on its implementation using a qualitative approach. Individual interviews with the participants and thematic analysis of verbatim transcripts were carried out. Ethics approval were obtained from the Research Ethics Committee on Human Subjects of the funding agency before the commencement of the study.

### The development of the integrative learning program

Delphi technique was employed to gain consensus in the best possible therapeutic interventions for old people with dementia among members of the expert panel^(27-30)^. The panel consisted of 8 health professionals including a gerontic care nurse, a registered nurse, an occupational therapist, a physiotherapist, a Chinese medicine practitioner, a mindfulness practitioner, a nutritionist, and a neuroscientist. They all had more than 10 years of experience in their own disciplines.

A member of our research team explained to the experts individually the purpose of having the panel and the importance of opinion sharing and idea exchange. Subsequent exchanges covered the conceptual framework, needs of people with dementia, components of the program operation and the intervention protocol. The experts did not meet but to provide their views through emails or audio recordings. We organized, collated the views, presented the aggregated views iteratively to each expert for consideration^(28)^. The exchange on each topic terminated when all experts reached total agreement on their analysis and views were exhausted. Upon completion of each round, a summary was sent to all experts for accuracy checking. With this iterative approach, 12 rounds of exchange were necessitated to develop the complete integrative learning protocol.

**Figure 1:**
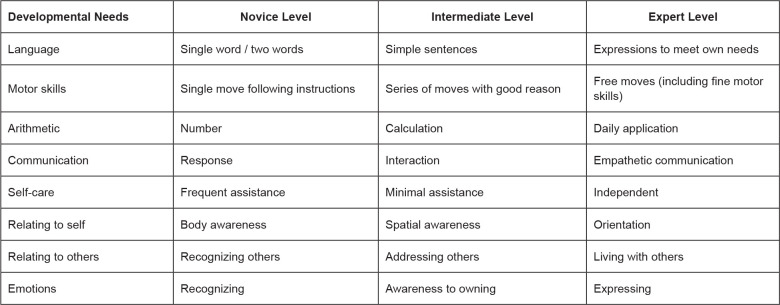
Needs and goals of development on a novice-expert continuum for people with dementia

To dispel any unrealistic expectations and facilitate learning, training in goal setting skills is needed in the integrative learning program. It was agreed among the experts that people with dementia have different needs and expectations, and various levels of difficulty and ability to meet them, be it basic (novice) or advanced (expert). Hence the panel formulated a list of developmental needs which are necessary and essential for quality living of people with dementia. The goal of development of each need was also identified based on a novice -expert continuum and the conceptual framework of neuroplasticity and learning (Figure 1).

The panel reviewed some common dementia management programs used in local institutions. Although these programs have included many contemporary treatment modalities, the results were not marked. The panel also commented that the treatment modalities could have been sufficient but the piece-meal approach might undermine their efficacy. They concluded that an integrated and structured multi-modality approach together with emotion management should be adopted for the integrative learning program. The multi-modality treatments in the program were structured sessions that employed physical, cognitive, social, and emotional stimulations to intervene dementia. The modalities selected for the program included reality orientation, daily living skills training, reminiscence therapy, multisensory stimulation, fall prevention program, mindfulness activities, Meridian exercise, brain health program (Four Arts of the Chinese Scholar which referred to zither, go, calligraphy and painting) and health education. It was also agreed that case managers, caregivers, and participants were free to choose these treatments or could be assigned to a treatment regime on a “mix and match” basis to suit their personality traits for improving their abilities to meet their own needs.

**Figure 2:**
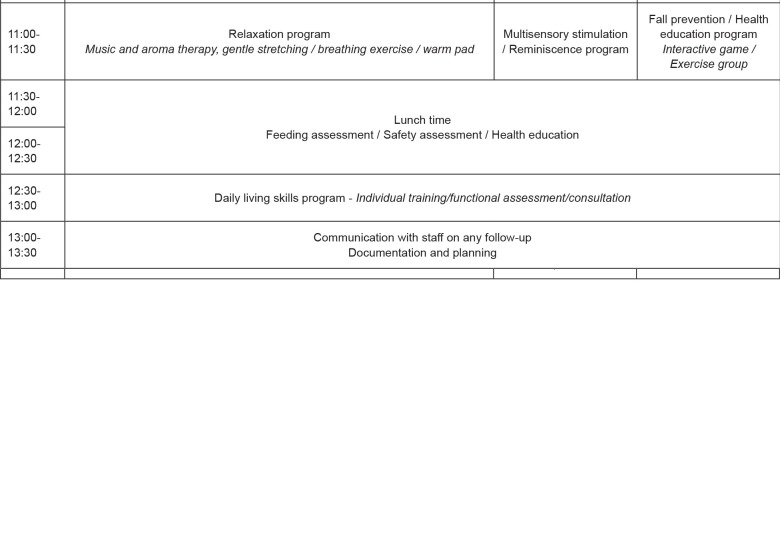
The 3-day integrative learning protocol

Based on the outcome of the above Delphi exercise, the experts brain-stormed the contents and details of each, followed by discussions before resolutions were made by consensus. Consensus was reached for the duration of a standard 3-day protocol and 5-day protocol. The 5-day protocol was an extended version of the 3-day one, which allowed the participants to practice more in a designated time. Figure 2 shows the 3-day integrative learning protocol. Interventions using the protocol were carried out by case managers who could be nurses, occupational therapists, or other health professionals. These case managers were independent of our research team. To ensure the competency of the case managers, each one had to attend a 3-day training workshop, with one day on theories, another day on practical training by 3 members of the Expert Panel (the gerontic care nurse, the nurse, and the occupational therapist) and the final day on quality assurance.

### Feasibility testing of the integrative learning program using a qualitative approach

The feasibility test which lasted for 6 months was held in community care centers run by the same non-profit organization (NPO) and their routine and care provided were the same across their centers. The centers were located in different districts where residents were in public housing. Two centers were randomly drawn from the NPO by drawing lots. Eligible older people with dementia from the centers were recruited by convenience sampling. The inclusion criteria were people aged 65 or above, members of the community care centers, diagnosed with dementia and mild cognition impairment (determined by the Montreal Cognitive Assessment Test for Dementia, MoCA score 12.7-20.1 depending on education level)^(31)^. Those living alone, with communication difficulties like language barriers, deafness, dysphasia and/or severe dysarthria, recently participated in another dementia program, with known history of psychotic illnesses such as schizophrenic, psychiatric, or with organic brain diseases such as brain tumors were excluded. All participants continued with their daily routines and medications during the research period. Consents were obtained from both the eligible older people and their relatives.

Qualitative data were based on the verbatim transcripts of the monthly case conferences. The verbatim transcripts of the case conferences were used for thematic analysis. These verbatim records were transcribed and coded for content analysis, based on the conceptual framework of the integrative learning program. A research assistant first identified words/segments in each transcript and then condensed them into meaningful units. Two members of our research team abstracted all the condensed meaning units into subthemes which were subsequently verified by three external experts.

## Results

The result of the integrative learning program which was reported above for easy reference. The protocol developed from the program was closely followed by the case managers and there was no indication for change throughout the implementation period.

The demographic data showed no significant differences in gender and age between the 2 centers. But there were statistically significant differences in educational level (p = 0.006) and duration of dementia (p = 0.001) between the 2 centers (Table 1).

**Table 1:** The demographic information of the participants by center. Hong Kong, 2018 (N=57)

Demographic Factor	Center 1*			Center 2*			Total			
*Sex* ^†^	*n*	*%*		*N*	*%*		*n*	*%*	*Z*	*p-value*
Female	34	68.0		5	71.4		39	68.4	0.033	1.000
Male	16	32.0		2	28.6		18	31.6		
*Age* ^‡^	*n*	*Mean (SD)*		*N*	*Mean (SD)*		*n*	*Mean (SD)*	*Z*	*p-value*
	39	85.1 (6.59)		6	84.7 (8.71)		45	85.0 (6.79)	-0.084	0.933
*Education Level*	*n*	*%*		*n*	*%*		*n*	*%*	*Z*	*p-value*
Non-educated	25	65.8		2	28.6		27	60.0	12.346	0.006§
Primary	13	34.2		3	42.9		16	35.6		
Secondary	0	0.0		1	14.3		1	2.2		
Post-secondary	0	0.0		1	14.3		1	2.2		
*Duration of Diagnosis*	*n*	*%*		*n*	*%*		*n*	*%*	*Z*	*p-value*
3 Months	11	22.0		0	0.0		11	19.3	14.763	0.001§
5 Months	26	52.0		0	0.0		26	45.6		
11 Months	13	26.0		7	100.0		20	35.1		

* Center 1 is residential center and Center 2 is a day-care center;

† Fisher’s Exact Test was used due to there were cells with expected count less than 5;

‡ Mann-Whitney U Test was used due to small sample size;

§ All p-values were considered as statistically significant when p 0.05

**Figure 3:**
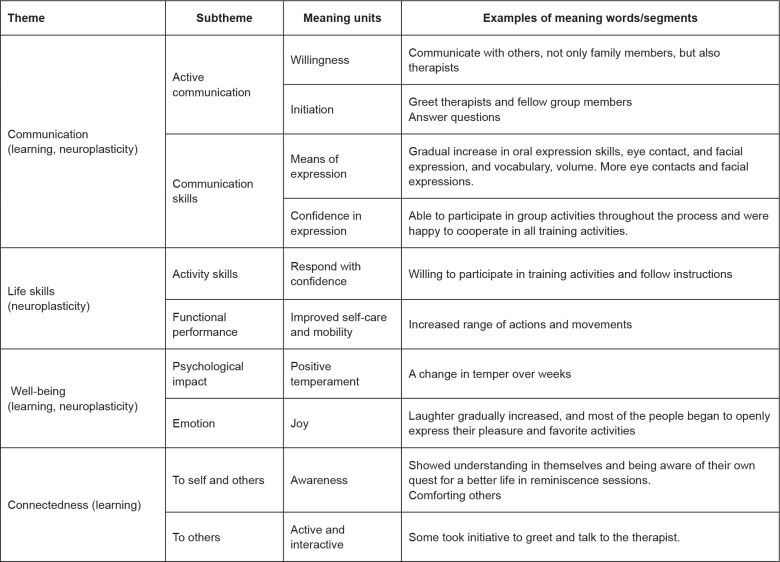
Content analysis of the case conferences. Hong Kong, 2018

A total of twelve verbatim records were collected, six from each center. To protect privacy of those not in the study, the team only studied those information related to the participants. With reference to the conceptual framework, communication and well-being were related to neuroplasticity and learning, and life skills reflected neuroplasticity while connectedness reflected mindful learning. Figure 3 shows the results of the content analysis. To ensure the trustworthiness of the analytic process, the team had invited three experienced aged care practitioners (each with at least 5 years of experience in aged care) to join in as content experts to verify the results after the subthemes were identified. They discussed the contents, subthemes and themes until they reach a consensus.

## Discussion

The participants showed obvious behavioral change which provided an insightful perspective on the effects of the program qualitatively. At the early stages of the program, some participants often left early or frequently asked to go to the bathroom and this situation improved significantly after they had been in contact with the case manager several times. Most participants were able to participate in group activities throughout the process and were happy to cooperate with all training activities. This behavioral change supports that people with dementia have the ability or the desire for self-improvement.

The participants over the 6-month period were increasingly more willing to communicate with others, not only with family members, but also with the case managers, their peers, and the institutional staff. Moreover, they began to use a wider range of oral expression skills with more vocabularies. This was in line with the idea advocated by UK’s National Health Service to encourage people with cognitive dysfunction to communicate with others as much as possible in order to slow down the impact of dementia. All participants in the 3-day and 5-day programs showed greater confidence in expressing themselves, using eye contacts, giving facial expressions, and raising their voice volume when answering questions.

After the integrative learning program intervention, most of the participants in the two centers were more confident, more reactive, and had an increased range of actions when doing lower limb exercises. Their willingness to participate in training activities and follow instructions had also improved over time. Similarly, their functional performance (e.g., self-care and mobility) was reported to have improved. The team believes that if this “guidance or encouragement” is not allowed, it affects the overall assessment of functional performance because “guidance or encouragement” is what this group of older people need.

A number of factors lowered the participation rates of some participants, which affected the results. For instance, some participants were unable to join some activities due to infection that required isolation. During an influenza outbreak in the program period, some group activities were restricted and, as a result, the affected participants could only attend personal practice sessions instead. In order to complete all sessions as scheduled, the actual participation time of those affected participants had to be shortened and therefore, could have influenced their performance in the program.

At the early stages of the intervention, the participants showed difficulty in controlling their temper. Their emotional expressions, somehow, drew the attention of the case managers (selected from therapists) who, on reflection, allowed them to make timely adjustments to related activities to better suit the participants’ needs. As time went on, laughter in the function room gradually increased, and most of the participants began to express their pleasure and preference in the running of the activities, and established a trusting therapeutic relationship with the case managers and a good rapport was developed between the two parties.

The increase in the participants’ pleasure could also be reflected in the verbatim. In the reminiscence sessions, the participants were gradually more willing to share details of their past with others, like recalling where they came from, how they spent their childhood, what their favorite food was, what things impressed them most, and so on, prompting them to start thinking for themselves and being aware of their own quest for a better life. The reminiscence therapy was very effective in an event before the Mid-Autumn festival. The participants were very eager to guess the prices of a variety of mooncakes. Some initiated to compare the prices with those in their old days and to evaluate whether the mooncakes were worth buying. In addition, some participants even took initiative to share the old practice of making monthly instalments to “Moon Cake Club” due to the low wages in the past, the fading out of “Moon Cake Club” etc.

Most of the participants became more active and interactive in the activities, and some out of their own volition began to greet and talk to the case managers, call out the case managers by name correctly, and gradually engage in some daily conversations, which did not occur in the early part of the intervention.

The integrative learning program focused on enhancing the participants’ abilities to implement whole-person care so as to enable institutional caregivers to better meet their needs. It was set up to use physical, cognitive, social, and emotional interventions, as well as some repetitive intervention plans. The repetitive intervention plans focused on developing the elderly’s sense of familiarity, security, and abilities in order to increase their participation, learning and development in all their functional areas. Case managers were sensitive to any possible plateau effects that participants might have and, thus, changed the intensity or type of activity components in the affected training sessions.

The program emphasizes self-determination, goal setting and sense of control. The team believes that people must have an understanding of their “self” before they can manage their dementia. The self of a person is manifested in the form of behaviors which evolve from interactions of different personality traits. In the case of the old people, these personality traits would have been shaped to a large extent by their life experiences and have been quite solidly grounded. Their understanding of their self is reflected through their behaviors. Therefore, the key to providing good care to people with dementia is to have a shared understanding of mutually acceptable goals among the case managers, caregivers and the old people concerned. This shared understanding means everyone needs to take an active role in the program, not just the recipients of care - the old people^(15)^. Also, it is crucial that the case managers, caregivers and the old people have the same understanding of the old people’s “self”. This common understanding helps formulate mutually acceptable goals. In this model, people with dementia were the active members in the center of care, while team members shared the roles and responsibilities of caregiving. Transdisciplinary care adopted an approach where case managers were chosen from a team of nurses, occupational therapists, physiotherapists, TCM physicians, western doctors, and pain management specialists basing on care needs.

Given the positive experience that we had in this methodological study, we have committed to actively conduct a multi-center trials to fine-tune the implementation of this transdisciplinary care program so as to benefit more people with dementia. More importantly, this study opens an alternative perspective in dementia care for researchers and those with dementia and their carers.

## Conclusion

This methodological study revealed the feasibility of an integrative approach in the care of old people with dementia. Dementia is considered as a global epidemic with huge potential costs. It is stereotyped by its progressive and irreversible impacts on individual’s health and wellbeing. The preliminary work reported in this paper has shed light on the possibility of rehabilitating old people with dementia through the integrative learning program developed from the theories of neuroplasticity and learning. Some positive effects of the program reflected from the results of the observations and judgments of the therapists were evidenced in case conferences. Given this is only a feasibility testing, the research team considered more robust and full scale evaluation of the program necessary and essential for better acceptance. The integrative program may exemplify the contributions of nursing to meet health needs where the demand is growing using a nurse-led model of care.

## References

[1] World Health Organization (2017). Global action plan on the public health response to dementia 2017 - 2025
[Internet].

[2] Chen Z, Yang X, Song Y, Song B, Zhang Y, Liu J (2017). Challenges of Dementia Care in China. Geriatrics.

[3] Wu YT, Ali GC, Guerchet M, Prina AM, Chan KY, Prince M (2018). Prevalence of dementia in mainland China, Hong Kong and Taiwan:
an updated systematic review and meta-analysis. Int J Epidemiol.

[4] World Health Organization (2018). Towards a dementia plan: A WHO guide [Internet].

[5] GBD 2016 Dementia Collaborators (2019). Global, regional, and national burden of Alzheimers disease and
other dementias, 1990-2016: a systematic analysis for the Global Burden of
Disease Study 2016. Lancet Neurol.

[6] Canadian Nurses’ Association (2016). Dementia in Canada: Recommendations to support care for Canada’s Aging
population: Brief prepared for the Senate Standing Committee on Social
Affairs, Science and Technology [Internet].

[7] Wimo A, Guerchet M, Ali GC, Wu YT, Prina AM, Winblad B (2017). The worldwide costs of dementia 2015 and comparisons with
2010. Alzheimers Dement.

[8] Song Y, Wang J (2010). Overview of Chinese research on senile dementia in mainland
China. Ageing Res Rev.

[9] Charidimou A, Viswanathan A (2016). Multiple neuropathologies and dementia in the aging brain: A key
role for cerebrovascular disease?. Alzheimers Dement (N Y).

[10] Shen Y, Ye B, Chen P, Wang Q, Fan C, Xiang M (2018). Cognitive Decline, Dementia, Alzheimer’s Disease and Presbycusis:
Examination of the Possible Molecular Mechanism. Frontiers Neurosci.

[11] Gratwicke J, Jahanshahi M, Foltynie T (2015). Parkinson’s disease dementia: a neural networks
perspective. Brain.

[12] Humpel C (2011). Chronic mild cerebrovascular dysfunction as a cause for
Alzheimers disease?. Exp Gerontol.

[13] Freund-Levi Y, Jedenius E, Tysen-Bäckström AC, Lärksäter M, Wahlund L, Eriksdotter M (2014). Galantamine versus Risperidone treatment of neuropsychiatric
symptoms in patients with probable dementia: An open randomized
trial. Am J Geriatr Psychiatry.

[14] Hainsworth AH, Yeo NE, Weekman EM, Wilcock DM (2016). Homocysteine, hyperhomocysteinemia and vascular contributions to
cognitive impairment and dementia (VCID). Biochim Biophys Acta.

[15] Bosco A, Schneider J, Coleston-Shields DM, Orrell M (2019). Dementia care model: Promoting personhood through
co-production. Arch Gerontol Geriatr.

[16] Lillard AS, Erisir A (2011). Old dogs learning new tricks: Neuroplasticity beyond the juvenile
period. Dev Rev.

[17] Lunghi C, Sale A (2015). A cycling lane for brain rewiring. Curr Biol.

[18] Caruso C (2016). New Experiences Help Speed Up Brain Development in Mice:
Researchers unravel how new neurons connect to existing neural networks
[Internet]. Scientific American.

[19] Särkämö T, Ripollés P, Vepsäläinen H, Autti T, Silvennoinen HM, Salli E (2014). Structural Changes Induced by Daily Music Listening in the
Recovering Brain after Middle Cerebral Artery Stroke: A Voxel-Based
Morphometry Study. Front Hum Neurosci.

[20] Marzouk S (2017). S182 Introduction to neuroplasticity and its application in
neurorehabilitation. Clin Neurophysiol.

[21] Adlaf EW, Vaden RJ, Niver AJ, Manuel AF, Onyilo VC, Araujo MT (2017). Adult-born neurons modify excitatory synaptic transmission to
existing neurons. eLife.

[22] Manuello J, Vercelli U, Nani A, Costa T, Cauda F (2016). Mindfulness meditation and consciousness: An integrative
neuroscientific perspective. Conscious Cogn.

[23] Dahlgaard J, Jørgensen MM, Velden AMVD, Sumbundu A, Mehlsen MY, Rattan SIS, Kyriazi M (2019). Mindfulness, Health, and Longevity. The Science of Hormesis in Health and Longevity.

[24] Langer EJ (2000). Mindful learning. Curr Dir Psychol Sci.

[25] Wang X, Geng L, Zhou K, Ye L, Ma Y, Zhang S (2016). Mindful learning can promote connectedness to nature: Implicit
and explicit evidence. Conscious Cogn.

[26] Tang Y, Geng L, Schultz PW, Zhou K, Xiang P (2017). The effects of mindful learning on pro-environmental behavior: A
self-expansion perspective. Consc Cogn.

[27] Mcpherson S, Reese C, Wendler MC (2018). Methodology Update: Delphi studies. Nurs Res.

[28] Flostrand A (2017). Finding the future: Crowdsourcing versus the Delphi
technique. Bus Horiz.

[29] Hasson F, Keeney S (2011). Enhancing rigour in the Delphi technique research. Technol Forecast Soc Change.

[30] Rowe G, Wright G (2011). The Delphi technique: Past, present, and future prospects —
Introduction to the special issue. Technol Forecast Soc Change.

[31] Wong A, Au LWC, Mok VCT, Tang AKY (2018). What’s in the web for family physicians - mild cognitive
impairment and dementia. Hong Kong Pract. [Internet].

